# Stem cell therapy for COVID-19 treatment: an umbrella review

**DOI:** 10.1097/JS9.0000000000001786

**Published:** 2024-07-05

**Authors:** Chaozhi Tang, Arkadiusz Dziedzic, Mahalaqua Nazli Khatib, Saad Alhumaid, Lakshmi Thangavelu, RP Parameswari, Prakasini Satapathy, Quazi Syed Zahiruddin, Sarvesh Rustagi, Maha Afri Alanazi, Majid S. Al-Thaqafy, Ali Hazazi, Jawaher Alotaibi, Nehad J. Al Faraj, Nisreen A. Al-Zaki, Mona J. Al Marshood, Thuria Y. Al Saffar, Khadija A. Alsultan, Shamsah H. Al-Ahmed, Ali A. Rabaan

**Affiliations:** a College of Life Science, Henan Normal University, Xinxiang, Henan, China; b Department of Conservative Dentistry with Endodontics, Medical University of Silesia, Katowice, Poland; c Division of Evidence Synthesis, Global Consortium of Public Health and Research, Datta Meghe Institute of Higher Education; d South Asia Infant Feeding Research Network (SAIFRN), Division of Evidence Synthesis, Global Consortium of Public Health and Research, Datta Meghe Institute of Higher Education, Wardha; e Center for Global Health Research, Saveetha Medical College and Hospital, Saveetha Institute of Medical and Technical Sciences, Saveetha University, Chennai; f School of Applied and Life Sciences, Uttaranchal University, Dehradun, Uttarakhand, India; g School of Pharmacy, University of Tasmania, Hobart, Australia; h Medical Laboratories Techniques Department, AL-Mustaqbal University, Hillah, Babil, Iraq; i Research Center, Prince Sultan Malitary Medical City, Riyadh; j Infection Prevention and Control Department, King Abdulaziz Medical City, National Guard Health Affairs; k Epidemiology and Public Health, King Abdullah International Medical Research Center, National Guard Health Affairs; l College of Medicine, King Saud Bin Abdulaziz University for Health Sciences, National Guard Health Affairs, Jeddah; m Department of Pathology and Laboratory Medicine, Security Forces Hospital Program, Riyadh, Saudi Arabia; n College of Medicine, Alfaisal University, Riyadh, Saudi Arabia; o Infectious Diseases Unit, Department of Medicine, King Faisal Specialist Hospital and Research Center, Riyadh; p Specialty Pediatric Medicine, Qatif Central Hospital, Qatif; q Molecular Diagnostic Laboratory, Johns Hopkins Aramco Healthcare, Dhahran, Saudi Arabia; r Department of Public Health and Nutrition, The University of Haripur, Haripur, Pakistan

**Keywords:** COVID-19, evidence-based medicine, global health, health systems resilience, stem cell therapy, sustainable development goals (SDG)

## Abstract

**Background:**

COVID-19 has presented significant obstacles to healthcare. Stem cell therapy, particularly mesenchymal stem cells, has emerged as a potential treatment modality due to its immunomodulatory and regenerative properties. This umbrella review aims to synthesize current evidence from systematic reviews on the safety and efficacy of stem cell therapy in COVID-19 treatment.

**Methods:**

A thorough literature search was performed across Embase, PubMed, Cochrane, and Web of Science from December 2019 to February 2024. Systematic reviews focusing on the use of stem cell therapy for COVID-19 were included. Evidence was synthesized by meta-analysis using R software (V 4.3) for each outcome. The certainty of evidence was assessed using the GRADE approach.

**Results:**

A total of 24 systematic reviews were included. Stem cell therapy was associated with reduced mortality [risk ratio (RR) 0.72, 95% CI: 0.60–0.86]; shorter hospital stays (mean difference −4.00 days, 95% CI: −4.68 to −3.32), and decreased need for invasive ventilation (RR 0.521, 95% CI: 0.320–0.847). Symptom remission rates improved (RR 1.151, 95% CI: 0.998–1.330), and a reduction in C-reactive protein levels was noted (standardized mean difference −1.198, 95% CI: −2.591 to 0.195), albeit with high heterogeneity. For adverse events, no significant differences were found between stem cell therapy and standard care (RR 0.87, 95% CI: 0.607–1.265). The certainty of evidence ranged from low to moderate.

**Conclusion:**

Stem cell therapy demonstrates a potential benefit in treating COVID-19, particularly in reducing mortality and hospital stay duration. Despite these promising findings, the evidence is varied, and future large-scale randomized trials are essential to confirm the efficacy and optimize the therapeutic protocols for stem cell therapy in the management of the disease. The safety profile is encouraging, with no significant increase in adverse events, suggesting a viable avenue for treatment expansion.

## Introduction

Highlights

- Stem cell therapy potentially reduces COVID-19 mortality, hospital stays, and invasive ventilation needs.
- Safety profile encouraging; no significant increase in adverse events compared to standard care.
- Evidence suggests the necessity for large-scale randomized controlled trials to confirm findings and optimize treatment protocols.
- Dual functionality modulates immune responses and promotes tissue regeneration, offering a comprehensive COVID-19 treatment strategy.
- Synthesizes evidence on stem cell therapy’s promising direction for COVID-19 mortality and morbidity reduction.

Stem cell therapy, at the forefront of regenerative medicine, has emerged as a potential therapeutic intervention for a range of diseases^1–5^, including COVID-19, instigated by the outbreak of severe acute respiratory syndrome coronavirus 2 (SARS-CoV-2)^6,7^. The pandemic has precipitated unparalleled challenges to global healthcare infrastructures, economies, and societal structures^8–11^. Stem cell therapy for spinal cord injury highlights their potential to modulate inflammation and promote tissue repair, offering insights that could improve treatment strategies for COVID-19-related complications^12^. The pathophysiology of COVID-19, particularly in its severe form, is characterized by an exacerbated immune response, culminating in significant tissue damage and underscoring the imperative for effective interventions that can modulate this response^13–15^. Despite progress in vaccine development and antiviral therapies, the quest for efficacious treatments remains critical, especially for severe cases^16–18^.

Within this context, stem cell therapy has garnered substantial interest for its unique capacity to modulate immune responses, promote tissue regeneration, and mitigate inflammation^19–21^. Integral to regenerative medicine, this therapeutic strategy involves the administration of stem cells to repair or replace damaged tissues. Among the various types of stem cells, mesenchymal stem cells (MSCs) have been extensively studied in the context of COVID-19 treatment due to their immunomodulatory properties, ability to produce anti-inflammatory cytokines, and potential to facilitate tissue repair^12,22,23^. The rationale for investigating stem cell therapy is rooted in the dual functionality of stem cells: their capability to regulate the immune system’s reaction and their capacity for tissue regeneration. Central to the severity of COVID-19 is the phenomenon known as the cytokine storm, a hyperactive immune response characterized by the excessive production of inflammatory cytokines, leading to widespread tissue damage, acute respiratory distress syndrome (ARDS), and other severe complications^24,25^. MSCs are posited as a viable therapeutic option due to their capabilities in modulating the immune response and reducing inflammation, potentially attenuating the cytokine storm linked with COVID-19 instances^26^. This could prevent or lessen the severity of ARDS and other complications, thereby improving patient prognoses. Furthermore, the potential of stem cells to differentiate into various cell types, including those constituting lung tissue, offers the possibility for the direct repair and regeneration of tissues affected by the virus, a critical aspect for patients experiencing lung damage^27–30^.

The application of stem cells in treating COVID-19 may expand our comprehension and management of viral infections and inflammatory diseases. Research into the effects of stem cell therapy on COVID-19 patients can provide critical insights into the mechanisms of immune modulation and tissue regeneration, potentially leading to innovative therapeutic approaches for a variety of conditions. Additionally, the ability of stem cell therapy to target both the immune response and tissue damage presents a unique therapeutic approach, distinguishing it from traditional antiviral or anti-inflammatory treatments. This positions stem cell therapy as a comprehensive treatment strategy for COVID-19, addressing both the etiological factors and the pathological consequences of the disease.

The rapidly expanding collection of studies on the use of stem cell therapy for treating COVID-19, including an array of systematic reviews, assesses the safety, efficacy, and clinical outcomes of stem cell treatment in COVID-19 patients, covering a wide spectrum of clinical indicators such as mortality rates, the necessity for mechanical ventilation, duration of hospital stays, and adverse events^31–36^. This umbrella review seeks to synthesize the evidence pertaining to stem cell treatment for COVID-19, identifying consistent findings, disparities, and existing gaps in knowledge. This approach provides a thorough insight into the current scope of research, emphasizing the level of evidence, potential benefits, and risks associated with stem cell therapy for COVID-19.

## Methods

This umbrella review was carried out following the Joanna Briggs Institute (JBI)^37^ methodology and adhered to the Preferred Reporting Items for Systematic Reviews and Meta-Analyses (PRISMA) guidelines^38^ (Supplementary Table S1, Supplemental Digital Content 1, http://links.lww.com/JS9/D45, Supplemental Digital Content 2, http://links.lww.com/JS9/D46). A protocol for this review has been prospectively registered in PROSPERO. For conducting this study, we utilized Nested Knowledge web software (Nested-Knowledge).

### Selection criteria

Systematic reviews, with or without meta-analyses, that have been published in peer-reviewed journals and offer a systematic evaluation of the effectiveness and safety of stem cell therapy in treating COVID-19 patients were included in the umbrella review. We targeted publications from the onset of the COVID-19 pandemic in December 2019 up to the date of the search, capturing the full range of emerging evidence during this global health crisis. The population criteria for the reviews included in this umbrella review are broad, encompassing patients diagnosed with COVID-19 irrespective of age, sex, disease severity, or the presence of comorbidities. In terms of intervention, the review focuses on studies evaluating the use of stem cell therapy in treating COVID-19. While MSCs are highlighted due to their prominent role in current research, the criteria are not limited to them alone. The outcomes of interest are clearly defined to include clinical endpoints critical in assessing the efficacy and safety of stem cell therapy. These include mortality rates, the necessity for mechanical ventilation, duration of hospital stays, improvements in lung function, and any reported adverse effects. Only reviews that synthesize data from rigorous study designs, such as randomized controlled trials (RCTs), case–control studies, non-RCTs, and cohort studies, are included. The language criterion limits the scope of the review to publications in English. Narrative reviews, editorials, opinion pieces, and letters are excluded to ensure a focus on systematic analysis or meta-analysis. Studies focusing solely on preclinical data, such as in vitro or animal studies without clinical outcomes, are also excluded, as the review aims to synthesize evidence directly applicable to human patients (Supplementary Table S2, Supplemental Digital Content 1, http://links.lww.com/JS9/D45).

### Literature search

A comprehensive literature search was performed across multiple databases, including Web of Science, Embase, PubMed, and Cochrane Library, for the period from 2019 to 15 February 2024. The search strategy involved combining terms related to ‘COVID-19’, ‘SARS-CoV-2’, and ‘coronavirus disease’ with those related to ‘stem cell therapy’ using Boolean operators (AND, OR). Keywords related to systematic review and meta-analysis were also used. No filters were applied regarding the type of article or language during the search. The detailed search strategy is displayed in Supplementary Table S3 (Supplemental Digital Content 1, http://links.lww.com/JS9/D45).

### Screening

The Nested Knowledge software was employed for the study selection process. Duplicates were removed prior to screening. The screening was performed by two independent researchers to minimize selection bias. Titles and abstracts were reviewed for alignment with the specified inclusion criteria. Full texts of potentially relevant reviews were then retrieved and assessed in detail for eligibility. Discrepancies between the reviewers were resolved by a third reviewer.

### Data extraction and quality assessment

The eligible systematic reviews were subjected to data extraction. Data including the author’s name, databases covered by the systematic review, number of RCTs included, year of RCTs, type of intervention, outcomes assessed, the tool used to assess the risk of bias, the findings from the risk of bias assessment, and any identified publication bias were extracted. For each outcome, effect sizes and their CIs were extracted based on each RCT included. Data extraction was performed using the ‘tagging’ function of Nested Knowledge software in a standardized data extraction form. The AMSTAR-2 tool was used for the quality assessment of the included systematic reviews^39^.

### Data synthesis

Results were synthesized for each outcome by performing a meta-analysis. Statistical computations were conducted in R statistical software, version 4.3^40–42^. For each outcome, a separate meta-analysis was performed by pooling the results from RCTs. For dichotomous outcomes, the number of events in the intervention and control groups, along with the number of participants in each group, were pooled to obtain the risk ratio (RR) and CI. Similarly, means and SD were used for outcomes that were reported as continuous. Heterogeneity was quantified by the *I*² statistic and Tau²^39,43,44^. A prediction interval was estimated. A *P*-value <0.05 was deemed to be statistically significant. Funnel plots were used for assessing publication bias^45^. The assessment of the level of certainty of evidence utilized the GRADE methodology (Grading of Recommendations, Assessment, Development, and Evaluations) system to evaluate the quality of evidence from the included systematic reviews and meta-analyses using GRADEpro Software^46^. The GRADE methodology categorizes evidence into four levels: high, moderate, low, and very low. High-quality evidence indicates that we are very confident that the true effect lies close to the estimate of effect, while moderate quality suggests moderate confidence where the true effect could be substantially different. Low-quality evidence implies limited confidence and very low-quality suggests that the true effect is likely to be substantially different from the estimate of effect. This classification is based on assessments of methodological flaws, inconsistencies, indirectness, imprecision, and potential publication bias within the underlying studies^47^.

## Results

### Search results

**Figure 1:**
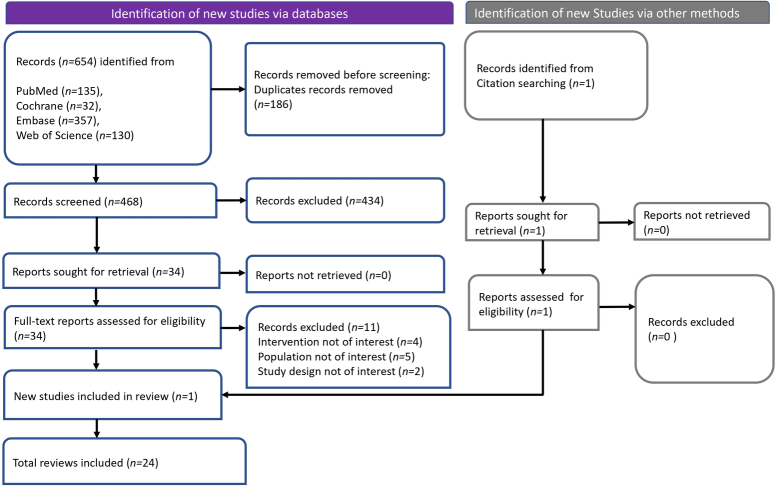
PRISMA flowchart illustrating the process of article screening and selection.

We identified a total of 654 records from databases. Prior to the screening process, we removed duplicate records, which accounted for 186 entries. Following the deduplication step, 468 records were screened, and a significant number of these, specifically 434 records, were excluded based on criteria that were not aligned with the objectives of the review. This left us with 34 reports that were deemed potentially relevant and were subsequently retrieved for a full-text assessment to evaluate their eligibility. In the full-text review phase, 11 reports were excluded for various reasons. Four reports were excluded because they involved interventions that were not of interest to our review, five were related to populations that did not meet our criteria, and two were omitted due to their study design being incompatible with our review objectives. Simultaneously, an additional search method via citation searching was employed, which yielded one record. This record was retrieved and assessed for eligibility, and as it met the inclusion criteria, it was included in the review. We identified one new study through database and register searching and one through citation searching. These were included in our review, bringing the total number of studies included in the systematic review to 24 (Fig. 1).

### Summary of included studies

The included systematic reviews, analyzing the impact of MSC therapy on COVID-19 treatment across several key outcomes between the year 2021 to 2023. A total of 24 systematic reviews were included^31–36,48–64^, with the number of included RCTs in each review ranging from 6 to 39, indicating a broad investigation of MSC therapy’s efficacy and safety in various contexts (Table 1). The interventions across these reviews were focused on MSCs, highlighting their potential in reducing mortality and morbidity, improving overall survival, and enhancing clinical outcomes in COVID-19 patients. Outcomes assessed included mortality rates, hospital stay lengths, inflammatory biomarker levels, and adverse events among others, showcasing a comprehensive evaluation of MSC therapy’s impact on COVID-19 treatment. The risk of bias within included RCTs was generally rated as moderate to high, reflecting the challenges in conducting research during a global health crisis. Most of the reviews suggested from these reviews suggest that MSC therapy can significantly lower mortality, improve survival rates without significantly affecting inflammatory markers, and is suggested as a safe treatment option for COVID-19. The certainty of evidence was not assessed in most of the included systematic review reviews. Supplementary Table S4 (Supplemental Digital Content 1, http://links.lww.com/JS9/D45) details the assessment results for the quality of the systematic reviews included in the study. Table 2 proved a summary of outcomes and their results with stem cell therapy.

**Table 1:** Characteristics of the included reviews.

Study ID	Databases and search	Number of included RCTs	Year range of included studies	Type of intervention	Outcomes	Risk of bias of included RCTs	Risk of bias tool used	Key findings
Arabpour 2021^48^	Pubmed/Medline, Embase, Scopus, Clinicaltrials.gov, and gray literature up to April 1, 2021	10	2020	Mesenchymal stem cells	Mortality, morbidity	Moderate	JBI tool for RCTs	Stem cell therapy is significantly effective in lowering mortality and morbidity in COVID-19 patients
Cao 2022^49^	PubMed, Cochrane Controlled Trials Register, EMBASE, and Web of Science, up to November 2021	13	2020	Mesenchymal stem cells	CRP, D-dimer, IL-6, overall survival (OS)	Moderate to High	Cochrane RoB	Mesenchymal stem cell therapy shows improvement in overall survival for COVID-19 patients without significantly affecting inflammatory markers
Chen 2022^50^	PubMed, Embase, Ovid MEDLINE, the Cochrane Library, and Clinicaltrials.gov, up to 7 November 2021	6	2020–2021	Mesenchymal stem cells	28-day overall mortality, clinical improvement rate, recovery time, hospital stay length, need for mechanical ventilation (MV), risk of AEs	Moderate to High	Cochrane RoB	MSC treatment is suggested to enhance clinical outcomes and is deemed a safe option for COVID-19 patients
Chen 2023^51^	PubMed, Embase, Cochrane Library, and Web of Science until 25 July 2022	8	2020–2022	Mesenchymal stem cells	Mortality risk, AEs, SpO_2_/FiO_2_, ICU stay	Moderate to Low	Cochrane RoB	Reduction in mortality among COVID-19 patients is observed with mesenchymal stem cell therapy
Couto 2023^52^	CellTrials.org, PUBMED, from January 2020 to December 2021	24	2020–2022	Mesenchymal stem cells	All-cause COVID-19 mortality	Not assessed	Not assessed	Advanced cell therapies as potentially crucial in treating COVID-19
Cuevas-Gonzalez 2021^53^	PUBMED, Scopus, and ScienceDirect, last search in March 2020	39	NA	Mesenchymal stem cells	ADRs	Low	Risk of bias for in vitro studies, the SciRAP tool	Shows stem cell therapy as a pivotal treatment, effectively counteracting cytokine secretion in both in vitro and clinical settings
Javed 2022^54^	PubMed, Embase, Scopus, CENTRAL, and clinicaltrials.gov up to December 31, 2021	22	2020–2021	Mesenchymal stem cells	Mortality, ADRs, biomarkers, O2 levels	Not assessed	Not assessed	Highlights the safety and tolerance of mesenchymal stem cells in COVID-19 patients, showing improvements in lung health and oxygenation while reducing viral and inflammatory indicators
Kandula 2023^55^	PubMed, EMBASE, Science Direct, Google Scholar, Scopus, Web of Science, and Cochrane Library until 31 December 2022	14	2020–2022	Mesenchymal stem cells	Mortality, ADRs	Moderate	Critical Appraisal Skills Program (CASP)	Affirms the promising role of MSC therapy in COVID-19 recovery, suggesting its routine application for complex conditions without adverse outcomes
Kirkham 2022^56^	MEDLINE and Embase and Cochrane Central Register of Controlled Trials, searched from 1947 up to 15 November 2021	11	2020–2021	Mesenchymal stem cells	Mortality, hospitalization, O2 levels, inflammatory biomarkers, ADRs	Moderate	Cochrane RoB-2	Early evidence suggests MSCs could reduce COVID-19 mortality, with a meta-analysis supporting decreased risk of death
Kirkham 2022 (2nd)^65^	MEDLINE and Embase and Cochrane, searched from 1947 up to 3 February 2021	9	2020	Mesenchymal stem cells	Mortality, interleukin 6	Moderate to High	Cochrane RoB-2	Indicates potential of MSC treatment in COVID-19 with caution due to few studies and detected risk of bias
Kirkham 2023^57^	MEDLINE, Embase and Cochrane, searched from 1947 through 30 March 2022	8	2020–2022	Mesenchymal stem cells	Comprehensive list including mortality, ICU admission, mechanical ventilation need, hospital/ICU stay duration, immune and inflammatory markers, AEs	Moderate	Cochrane RoB-2	MSCs are seen as potentially reducing death risk in severe COVID-19 cases and improving various clinical outcomes, though variable reporting and product characterization limit evidence quality
Li 2023^58^	PubMed, Embase, Cochrane, and Web of Science until 24th August 2021	8	Not available	Mesenchymal stem cells	AEs, SAEs, mortality rate	Not available	Cochrane RoB-2	Suggests promising safety and efficacy of MSC therapy in reducing COVID-19 mortality without increasing adverse events
Liu 2023^31^	Until 22 March 2022, the PubMed, EMBASE, Cochrane Library, Web of Science, and ClinicalTrials databases	7	2020–2022	Mesenchymal stem cells	Mortality, inflammatory biomarkers, effects on CRP, interferon-gamma, interleukin-6	Moderate to Low	Cochrane RoB	Finds umbilical cord-derived MSC infusion as an effective COVID-19 pneumonia treatment with no significant adverse effects observed
Qu 2022^59^	Databases included Cochrane Central Register of Controlled Trials, Ovid MEDLINE, and Scopus until 3 November 2021	34	2020–2021	Mesenchymal stem cells	All-cause mortality, SAEs	Moderate to Low	Cochrane RoB-2	Supports MSC cell therapy’s potential in decreasing all-cause mortality and SAEs, improving pulmonary function versus conventional care
Tamis 2023^32^	PubMed, Web of Science, and Google Scholar up to July 2022	10	2020–2022	Mesenchymal stem cells	Mortality	Moderate to high	Modified JADAD for RCTs, ROBINS-I for non-RCTs	Advocates for stem cell therapy as a safe and effective method to diminish death rates and boost pulmonary function in COVID-19 patients, alongside anti-inflammatory benefits
Taufiq 2023^60^	PubMed, Science Direct, BSCOhost, Google Scholar, The Cochrane Library, bioRxiv, and Clinical Trials.gov.up to 23 March 2022	8	2020–2022	Mesenchymal stem cells	Mortality, AEs, SAEs, CRP, IL-6 levels	Moderate to Low	Cochrane RoB-2	Indicates MSCs as safe with a lower mortality risk and no significant AEs, showing potential improvements in inflammatory response and lung function, though more research is needed for conclusive efficacy evidence
Wang 2021^33^	PubMed, Embase, Cochrane Library, WAN FANG, and CNKI databases. From October 2019 to April 2021 across	22	2021–2022	Allogeneic MSCs	AEs, mortality rate	Moderate to high	Cochrane RoB	Demonstrates the effectiveness and safety of MSC therapy in treating COVID-19 pneumonia, highlighting its role in reducing AEs and mortality
Wang 2023^34^	PubMed and EMBASE up to November 2022.	13	2018–2022	Mesenchymal stem cells	ADRs, mortality, hospitalization	Moderate to Low	Cochrane RoB	Suggests MSC administration in ARDS patients as safe and potentially beneficial in improving survival, though further evidence is needed for conclusive claims
Yan 2023^35^	China National Knowledge Infrastructure, CENTRAL, Embase, Web of Science, and PubMed, up to 30 April 2022	8	2020–2022	Mesenchymal stem cells	Mortality, clinical symptom improvement rate, C-reactive protein, days to hospital discharge	Moderate to Low	Cochrane RoB-1	Highlights MSCs as a feasible therapy for COVID-19, noting safety and potential efficacy in reducing mortality, accelerating clinical improvement, and lowering CRP levels, with no significant adverse reactions detected
Yang 2023^36^	Medline, Embase, PubMed, Cochrane Library, and Web of Science databases, up to 1 December 1, 2021	10	2020–2022	Umbilical cord MSCs	Mortality, adverse events	Low to Moderate	Modified JADAD for RCTs, ROBINS-I for non-RCTs	Positions UC-MSCs as a promising and safe treatment option for COVID-19, with an emphasis on its potential efficacy
Yao 2022^61^	PubMed, the Cochrane Library, and the Chinese electronic database CNKI were searched from inception up to 19 December 2021	14	2020–2021	Mesenchymal stem cells	In-hospital mortality, adverse events (AEs)	Moderate to High	Cochrane RoB-2, ROBINS-I	Confirms that MSCs likely reduce mortality and are safe for severe or critical COVID-19 patients, with dose variation not significantly impacting outcomes
Zanirati 2021^62^	MEDLINE (via PubMed), Embase, and Scopus from January 2000 to March 2021.	37	2014–2021	Mesenchymal stem cells	Lymphocytes, CRP level, Plasma cytokines, TNF alpha, PaO_2_/FiO_2_, AE, Mortality	Not assessed	Not assessed	Indicates a positive impact of stem cell therapy on key immunological and inflammatory processes in lung injury for COVID-19 and ARDS patients, with no therapy-related deaths reported among the studies
Zhang 2022^63^	PubMed, Embase, Web of Science, The Cochrane Library, CNKI, WanFang, VIP, and SinoMed up to 18 January 2022	17	2020–2021	Mesenchymal stem cells	AEs, SAEs, mortality rate, hospitalization	Moderate	Cochrane RoB	Shows stem cell therapy for COVID-19 as highly efficient without increasing adverse event risks or hospital stay lengths, suggesting the need for established criteria for stem cell therapy application in COVID-19
Zhang 2023^64^	PubMed, Embase, Cochrane Library, Web of Science, WanFang, and CNKI, up to 25 December 2022	17	2020–2022	Mesenchymal stem cells	All-cause mortality, symptom, remission rate, LOS, ICU days, oxygen therapy requirements, biomarkers of inflammatory dysregulation, and pulmonary imaging changes	Moderate to High	Cochrane RoB-2	MSC therapy may reduce all-cause mortality in COVID-19 patients without increasing risks of adverse events, although it may not significantly improve symptom remission rates. TSA analysis suggests caution in interpreting mortality benefits
Arabpour 2021^1^	Pubmed/Medline, Embase, Scopus, Clinicaltrials.gov, and gray literature up to 1 April 2021	10	2020	Mesenchymal stem cells	Mortality, morbidity	Moderate	JBI tool for RCTs	Stem cell therapy is significantly effective in lowering mortality and morbidity in COVID-19 patients
Cao 2022^2^	PubMed, Cochrane Controlled Trials Register, EMBASE, and Web of Science, up to November 2021	13	2020	Mesenchymal stem cells	CRP, D-dimer, IL-6, overall survival (OS)	Moderate to High	Cochrane RoB	Mesenchymal stem cell therapy shows improvement in overall survival for COVID-19 patients without significantly affecting inflammatory markers
Chen 2022^3^	PubMed, Embase, Ovid MEDLINE, the Cochrane Library, and Clinicaltrials.gov, up to 7 November 2021	6	2020–2021	Mesenchymal stem cells	28-day overall mortality, clinical improvement rate, recovery time, hospital stay length, need for mechanical ventilation (MV), risk of AEs	Moderate to High	Cochrane RoB	MSC treatment is suggested to enhance clinical outcomes and is deemed a safe option for COVID-19 patients
Chen 2023^4^	PubMed, Embase, Cochrane Library, and Web of Science until 25 July 2022	8	2020–2022	Mesenchymal stem cells	Mortality risk, AEs, SpO_2_/FiO_2_, ICU stay	Moderate to Low	Cochrane RoB	Reduction in mortality among COVID-19 patients is observed with mesenchymal stem cell therapy
Couto 2023^5^	CellTrials.org, PUBMED, from January 2020 to December 2021	24	2020–2022	Mesenchymal stem cells	All-cause COVID-19 mortality	Not assessed	Not assessed	Advanced cell therapies as potentially crucial in treating COVID-19
Cuevas-Gonzalez 2021^6^	PUBMED, Scopus, and ScienceDirect, last search in March 2020	39	NA	Mesenchymal stem cells	ADRs	Low	Risk of bias for in vitro studies, the SciRAP tool	Shows stem cell therapy as a pivotal treatment, effectively counteracting cytokine secretion in both in vitro and clinical settings
Javed 2022^7^	PubMed, Embase, Scopus, CENTRAL, and clinicaltrials.gov up to December 31, 2021	22	2020–2021	Mesenchymal stem cells	Mortality, ADRs, biomarkers, O_2_ levels	Not assessed	Not assessed	Highlights the safety and tolerance of mesenchymal stem cells in COVID-19 patients, showing improvements in lung health and oxygenation while reducing viral and inflammatory indicators
Kandula 2023^8^	PubMed, EMBASE, Science Direct, Google Scholar, Scopus, Web of Science, and Cochrane Library until 31 December 2022	14	2020–2022	Mesenchymal stem cells	Mortality, ADRs	Moderate	Critical Appraisal Skills Program (CASP)	Affirms the promising role of MSC therapy in COVID-19 recovery, suggesting its routine application for complex conditions without adverse outcomes
Kirkham 2022^9^	MEDLINE and Embase and Cochrane Central Register of Controlled Trials, searched from 1947 up to 15 November 2021	11	2020–2021	Mesenchymal stem cells	Mortality, hospitalization, O2 levels, inflammatory biomarkers, ADRs	Moderate	Cochrane RoB-2	Early evidence suggests MSCs could reduce COVID-19 mortality, with a meta-analysis supporting decreased risk of death
Kirkham 2022 (2nd)^10^	MEDLINE and Embase and Cochrane, searched from 1947 up to 3 February 2021	9	2020	Mesenchymal stem cells	Mortality, interleukin 6	Moderate to High	Cochrane RoB-2	Indicates potential of MSC treatment in COVID-19 with caution due to few studies and detected risk of bias
Kirkham 2023^11^	MEDLINE, Embase and Cochrane, searched from 1947 through 30 March 2022	8	2020–2022	Mesenchymal stem cells	Comprehensive list including mortality, ICU admission, mechanical ventilation need, hospital/ICU stay duration, immune and inflammatory markers, AEs	Moderate	Cochrane RoB-2	MSCs are seen as potentially reducing death risk in severe COVID-19 cases and improving various clinical outcomes, though variable reporting and product characterization limit evidence quality
Li 2023^12^	PubMed, Embase, Cochrane, and Web of Science until 24th August 2021	8	Not available	Mesenchymal stem cells	AEs, SAEs, the mortality rate	Not available	Cochrane RoB-2	Suggests promising safety and efficacy of MSC therapy in reducing COVID-19 mortality without increasing adverse events
Liu 2023^13^	Until 22 March 2022, the PubMed, EMBASE, Cochrane Library, Web of Science, and ClinicalTrials databases	7	2020–2022	Mesenchymal stem cells	Mortality, inflammatory biomarkers, effects on CRP, interferon-gamma, interleukin-6.	Moderate to Low	Cochrane RoB	Finds umbilical cord-derived MSC infusion as an effective COVID-19 pneumonia treatment with no significant adverse effects observed
Qu 2022^14^	Databases included Cochrane Central Register of Controlled Trials, Ovid MEDLINE, and Scopus until 3 November 2021	34	2020–2021	Mesenchymal stem cells	All-cause mortality, SAEs	Moderate to Low	Cochrane RoB-2	Supports MSC cell therapy’s potential in decreasing all-cause mortality and SAEs, improving pulmonary function versus conventional care
Tamis 2023^15^	PubMed, Web of Science, and Google Scholar up to July 2022	10	2020–2022	Mesenchymal stem cells	Mortality	Moderate to high	Modified JADAD for RCTs, ROBINS-I for non-RCTs	Advocates for stem cell therapy as a safe and effective method to diminish death rates and boost pulmonary function in COVID-19 patients, alongside anti-inflammatory benefits
Taufiq 2023^16^	PubMed, Science Direct, BSCOhost, Google Scholar, The Cochrane Library, bioRxiv, and Clinical Trials.gov.up to 23 March 2022	8	2020–2022	Mesenchymal stem cells	Mortality, AEs, SAEs, CRP, IL-6 levels	Moderate to Low	Cochrane RoB-2	Indicates MSCs as safe with a lower mortality risk and no significant AEs, showing potential improvements in inflammatory response and lung function, though more research is needed for conclusive efficacy evidence
Wang 2021^17^	PubMed, Embase, Cochrane Library, WAN FANG, and CNKI databases. From October 2019 to April 2021 across	22	2021–2022	Allogeneic MSCs	AEs, mortality rate	Moderate to high	Cochrane RoB	Demonstrates the effectiveness and safety of MSC therapy in treating COVID-19 pneumonia, highlighting its role in reducing AEs and mortality
Wang 2023^18^	PubMed and EMBASE up to November 2022	13	2018–2022	Mesenchymal stem cells	ADRs, mortality, hospitalization	Moderate to Low	Cochrane RoB	Suggests MSC administration in ARDS patients as safe and potentially beneficial in improving survival, though further evidence is needed for conclusive claims
Yan 2023^19^	China National Knowledge Infrastructure, CENTRAL, Embase, Web of Science, and PubMed, up to 30 April 2022	8	2020–2022	Mesenchymal stem cells	Mortality, clinical symptom improvement rate, C-reactive protein, days to hospital discharge	Moderate to Low	Cochrane RoB-1	Highlights MSCs as a feasible therapy for COVID-19, noting safety and potential efficacy in reducing mortality, accelerating clinical improvement, and lowering CRP levels, with no significant adverse reactions detected
Yang 2023^20^	Medline, Embase, PubMed, Cochrane Library, and Web of Science databases, up to 1 December 2021	10	2020–2022	Umbilical cord MSCs	Mortality, adverse events	Low to Moderate	Modified JADAD for RCTs, ROBINS-I for non-RCTs	Positions UC-MSCs as a promising and safe treatment option for COVID-19, with an emphasis on its potential efficacy
Yao 2022^21^	PubMed, the Cochrane Library, and the Chinese electronic database CNKI were searched from inception up to 19 December 2021	14	2020–2021	Mesenchymal stem cells	In-hospital mortality, adverse events (AEs)	Moderate to High	Cochrane RoB-2, ROBINS-I	Confirms that MSCs likely reduce mortality and are safe for severe or critical COVID-19 patients, with dose variation not significantly impacting outcomes
Zanirati 2021^22^	MEDLINE (via PubMed), Embase, and Scopus from January 2000 to March 2021	37	2014–2021	Mesenchymal stem cells	Lymphocytes, CRP level, Plasma cytokines, TNF alpha, PaO2/FiO2, AE, Mortality	Not assessed	Not assessed	Indicates a positive impact of stem cell therapy on key immunological and inflammatory processes in lung injury for COVID-19 and ARDS patients, with no therapy-related deaths reported among the studies
Zhang 2022^23^	PubMed, Embase, Web of Science, The Cochrane Library, CNKI, WanFang, VIP, and SinoMed up to 18 January 2022	17	2020–2021	Mesenchymal stem cells	AEs, SAEs, mortality rate, hospitalization	Moderate	Cochrane RoB	Shows stem cell therapy for COVID-19 as highly efficient without increasing adverse event risks or hospital stay lengths, suggesting the need for established criteria for stem cell therapy application in COVID-19
Zhang 2023^24^	PubMed, Embase, Cochrane Library, Web of Science, WanFang, and CNKI, up to 25 December 2022	17	2020–2022	Mesenchymal stem cells	All-cause mortality, symptom, remission rate, LOS, ICU days, oxygen therapy requirements, biomarkers of inflammatory dysregulation, and pulmonary imaging changes	Moderate to High	Cochrane RoB-2	MSC therapy may reduce all-cause mortality in COVID-19 patients without increasing risks of adverse events, although it may not significantly improve symptom remission rates. TSA analysis suggests caution in interpreting mortality benefits

ADRs, Adverse Drug Reactions; AEs, Adverse Events; CASP, Critical Appraisal Skills Program; CNKI, China National Knowledge Infrastructure; CRP, C-Reactive Protein; FiO_2_, Fraction of Inspired Oxygen; IL-6, Interleukin 6; JADAD, Jadad Scale for Reporting Randomized Controlled Trials; LOS, Length of Stay; MSCs, Mesenchymal Stem Cells; OS, Overall Survival; PaO_2_, Partial Pressure of Oxygen; RCTs, Randomized Controlled Trials; SAEs, Serious Adverse Events; TNF alpha, Tumor Necrosis Factor alpha; TSA, Trial Sequential Analysis; UC-MSCs, Umbilical Cord Mesenchymal Stem Cells.

**Table 2:** Summary of the outcomes and their results with stem cell therapy.

Outcome	Number of studies	Number of participants	Effect size (95% CI)	Heterogeneity (*I* ^2^)	Publication bias	GRADE
Mortality	33	1422	RR=0.722 (0.591–0.883)	0%	Not suspected	Low
CRP level	6	220	SMD= −1.198 (−2.591 to 0.195)	89%	NA	Low
Symptom remission rate	12	731	RR=1.151 (0.998–1.330)	28%	Not suspected	Very low
Length of hospitalization (Days)	5	269	MD= −4.00 (−4.68 to −3.337)	0%	NA	Moderate
Adverse events	17	860	RR=0.87 (0.607–1.265)	34%	Not suspected	Low
Serious adverse events	7	298	RR=0.899 (0.202–3.99)	43%	NA	Low
Mechanical invasive ventilation	4	328	RR=0.521 (320–0.847)	0%	NA	Moderate
Time for symptom improvement	2	99	MD=−4.01 (−6.33 to −1.68)	54%	NA	Low
Spo_2_/FiO_2_	4	163	WMD=−4.29 (−22.26 to 30.6)	93%	NA	Very low

CRP, C-Reactive Protein; FiO_2_, Fraction of Inspired Oxygen; GRADE, Grading of Recommendations, Assessment, Development, and Evaluations; MD, Mean Difference; NA, Not Available; RR, Relative Risk; SMD, Standardized Mean Difference; SpO_2_, Peripheral Capillary Oxygen Saturation; WMD, Weighted Mean Difference.

### Mortality

**Figure 2:**
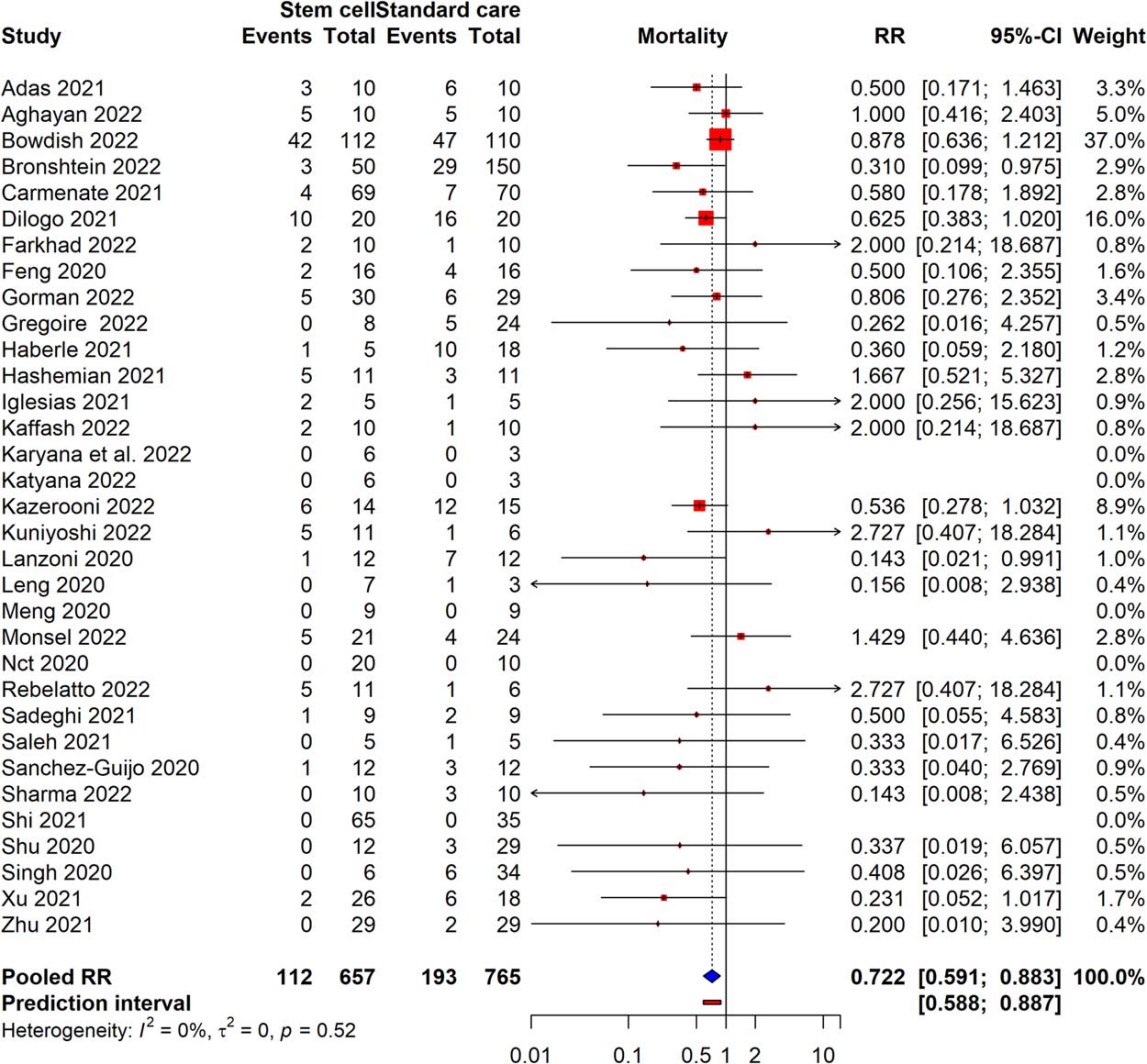
Forest plot of mortality rates in COVID-19 patients receiving stem cell therapy versus those receiving standard care.

From the meta-analysis of 33 studies with a total of 1422 COVID-19 patients, we found that stem cell therapy significantly reduced the mortality rate, with an RR of 0.72, indicating a 28% reduction in the risk of mortality for patients undergoing this treatment. The statistical significance of these findings was bolstered by a *P*-value of 0.002, and the absence of heterogeneity across studies (*I* ^2^=0%) highlighted the consistency of the therapy’s beneficial effects across various settings. Additionally, an Egger’s test *P*-value of 0.17 suggested no significant publication bias. A forest plot visualized in Figure 2 of our manuscript provides a detailed graphical representation of these findings. The certainty of evidence was found to be low (Supplementary Table S5, Supplemental Digital Content 1, http://links.lww.com/JS9/D45).

### Length of hospitalization

**Figure 3:**
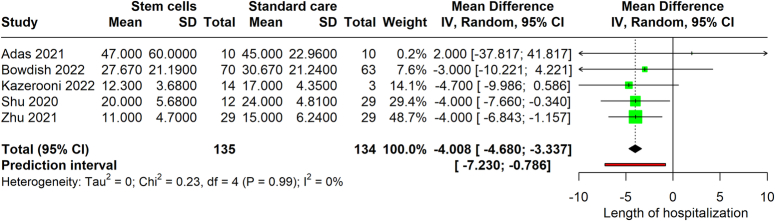
Forest plot comparing the length of hospital stay for COVID-19 patients treated with stem cell therapy against standard care.

We reviewed data from five studies, encompassing a total of 269 participants. Our research revealed a notable decrease in the duration of hospital admissions for individuals treated, emphasizing the potential efficacy of the investigated intervention in enhancing patient recovery times and optimizing healthcare resource utilization, with a mean difference (MD) of −4.00 (95% CI: −4.68 and −3.32, *P≤*0.0001). This indicates that, on average, the hospitalization duration for patients subjected to the stem cell therapy was 4 days shorter than for those who did not receive it. The 95% CI for this mean difference was tightly bound between −4.68 and −3.32 days, reinforcing the precision and reliability of this significant reduction. Furthermore, the heterogeneity among the included studies was nonexistent, as shown by an *I* ^2^ value of 0%, indicating that the effect of the intervention on reducing hospitalization length was consistent across different studies and populations. Figure 3 displays the results of a meta-analysis. The certainty of evidence was found to be moderate.

### Symptom remission rate

**Figure 4:**
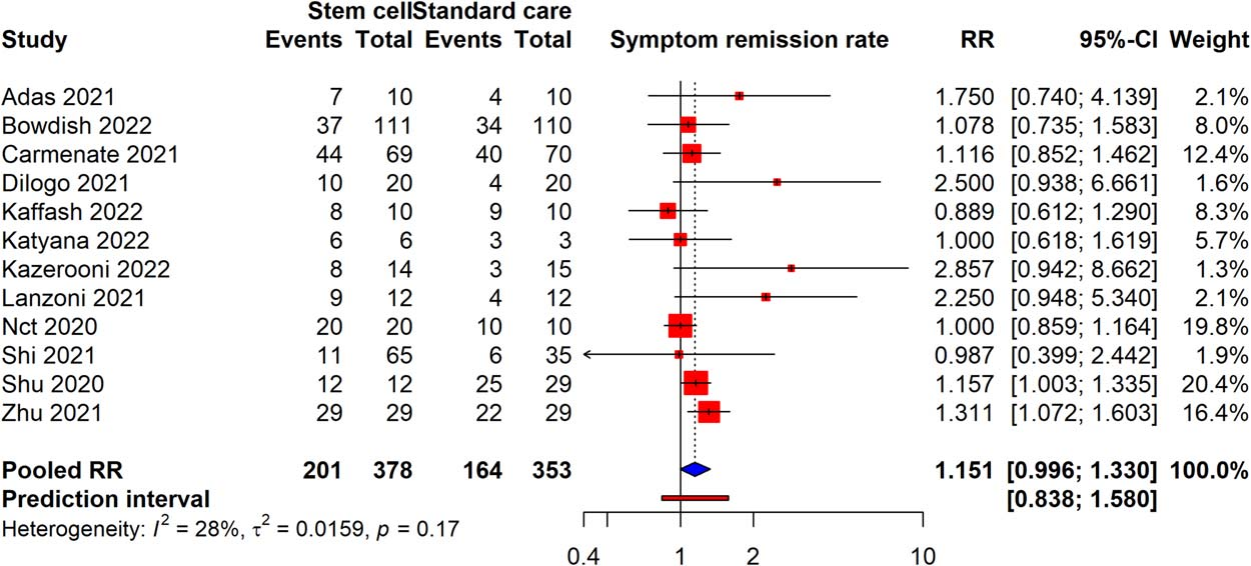
Forest plot analysis of symptom remission rates in COVID-19 patients treated with stem cell therapy versus standard care.

From 12 studies that included a total of 724 participants, the meta-analysis revealed that the stem cell therapy was co-related with an increased rate of symptom remission, as indicated by a RR of 1.151 (95% CI: 0.998–1.330, *P*=0.056). This suggests that patients undergoing the stem cell therapy were ~15% more likely to experience remission of symptoms compared to those who did not receive the intervention (Fig. 4). The 95% CI for this effect size ranged from 0.998 to 1.330, approaching statistical significance and indicating a trend towards effectiveness in promoting symptom remission. The heterogeneity among the studies was moderate, with an *I* ^2^ value of 28%. The presence publication bias was not suspected (Egger’s test, *P*=0.122). The evidence’s reliability was assessed to be very low.

### CRP level

**Figure 5:**
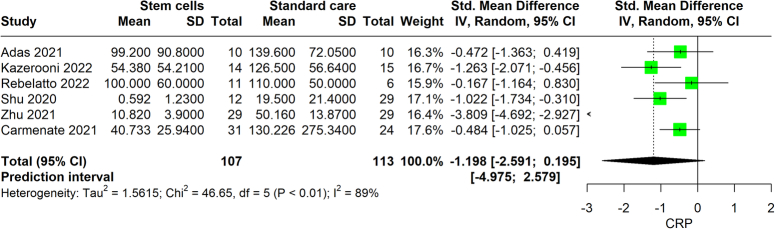
Forest plot comparison of C-reactive protein level changes in COVID-19 patients under stem cell therapy versus standard treatment.

From six studies involving a total of 220 participants, stem cell therapy was associated with a decrease in C-reactive protein (CRP) levels, indicative of reduced inflammation. The therapy yielded a standardized mean difference (SMD) of −1.198 (95% CI: −2.591 to 0.195, *P*=0.078), suggesting a decrease in CRP levels among patients who were treated with the stem cell therapy compared to those who did not. This indicates a potentially effective reduction in inflammation, despite the wide range of the CI, which still points towards a trend of clinically relevant CRP level reduction (Fig. 5). However, the analysis exposed a high degree of heterogeneity among the included studies, as evidenced by an *I* ^2^ value of 89%. The evidence’s reliability was assessed to be low.

### Need for invasive ventilation

**Figure 6:**
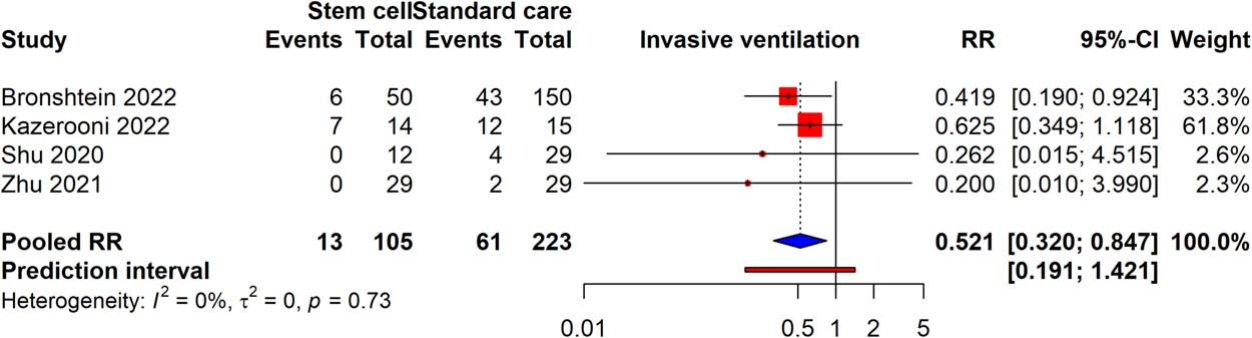
Forest plot evaluating the necessity for invasive ventilation in COVID-19 patients treated with stem cell therapy versus standard care.

From four studies involving 328 participants, stem cell therapy was found to significantly reduce the need for mechanical invasive ventilation, as shown by a RR of 0.521 (95% CI: 0.320–0.847, *P*=0.0235). This outcome shows a 47.9% decrease in the probability of requiring mechanical invasive ventilation for patients who received stem cell therapy compared to those who did not, highlighting the stem cell therapy’s potential effectiveness in treating severe cases of COVID-19, with heterogeneity of *I* ^2^=0% (Fig. 6). The evidence’s reliability was assessed to be moderate.

### SpO_2_/FiO_2_

**Figure 7:**
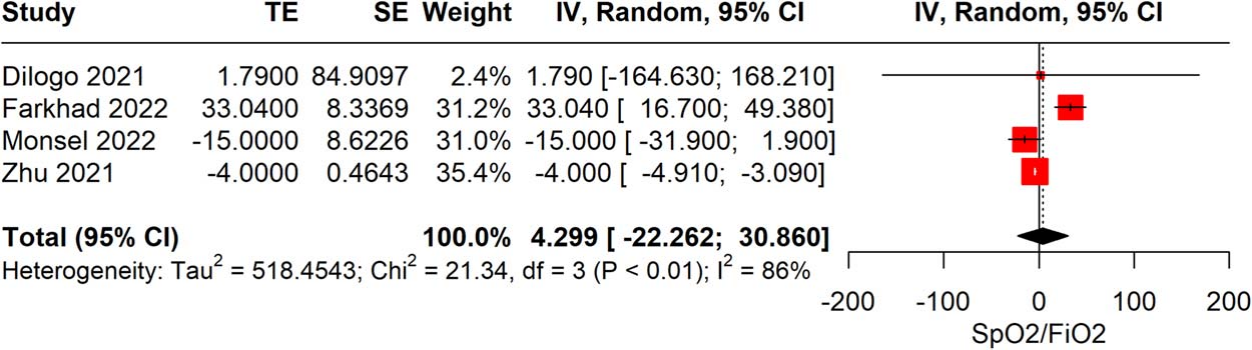
Forest plot comparison of SpO_2_/FiO_2_ ratio improvements in COVID-19 patients receiving stem cell therapy against those on standard care.

From the provided data on the impact of stem cell therapy on the SpO_2_/FiO_2_ ratio in COVID-19 patients, four studies with a total of 163 participants were analyzed. The intervention’s effect on improving the SpO_2_/FiO_2_ ratio, a measure of oxygenation efficiency, was not statistically significant, as indicated by a WMD of −4.29 (95% CI: −22.26 to 30.6), *P*=0.751. The lack of statistical significance, suggesting that the intervention does not have a notable impact on enhancing oxygenation in COVID-19 patients as measured by the SpO_2_/FiO_2_ ratio (Fig. 7). The analysis revealed a very high degree of heterogeneity (*I* ^2^=93%) among the included studies. The evidence’s reliability was assessed to be very low.

### Time for symptom improvement

Data from two studies involving a total of 99 participants were examined^36^. Stem cell therapy significantly reduced the time required for symptom improvement, with an MD of −4.01 days (95% CI: −6.33 to −1.68, *P*=0.03). The heterogeneity was found to be high (*I* ^2^=54). The certainty of evidence was found to be low.

### Adverse events

**Figure 8:**
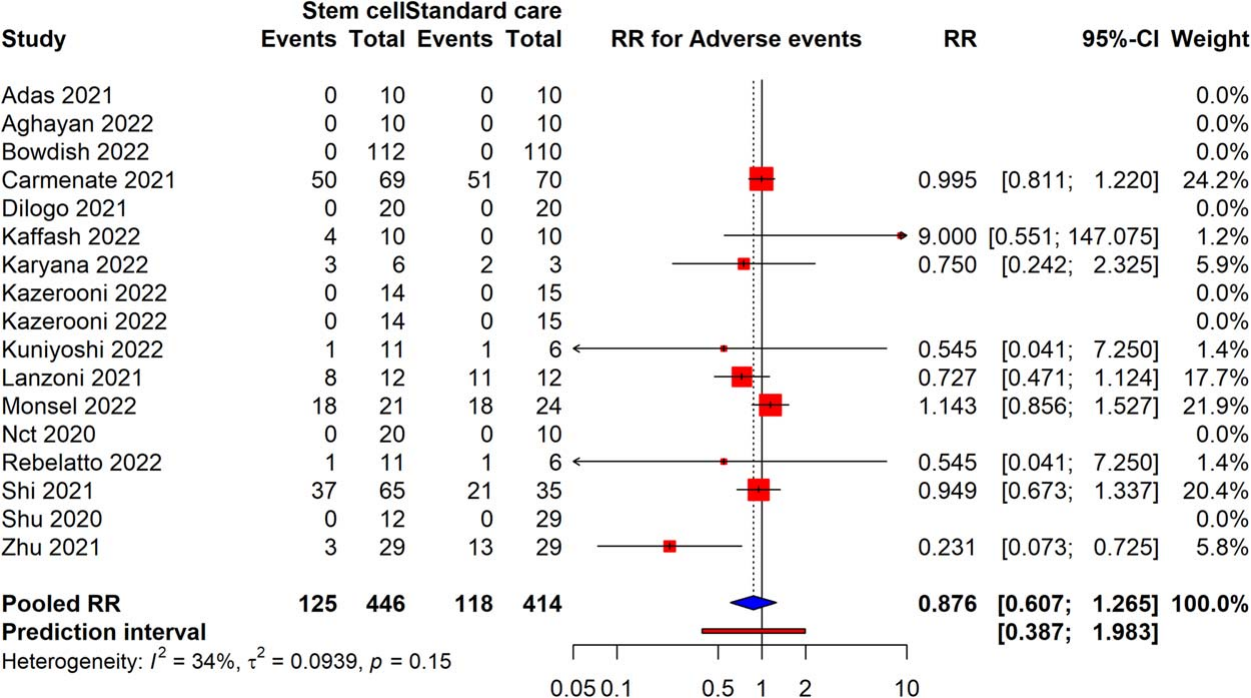
Forest plot assessing the rate of adverse events in COVID-19 patients receiving stem cell therapy versus standard care.

For adverse events, data from 17 studies involving 860 participants (446 in the intervention group and 414 in the control group) were analyzed. The stem cell therapy showed a RR of 0.87 (95% CI: 0.607–1.265), indicating a nonsignificant reduction in the occurrence of adverse events among patients receiving the intervention compared to those who did not (Fig. 8). The CI suggests there is considerable uncertainty around the effect size, and the *P*-value was not provided to quantify the statistical significance. The moderate heterogeneity among studies (*I* ^2^=34%) indicates some variability in the intervention’s impact on adverse events across different study designs and patient populations but is not excessively high. The evidence’s reliability was assessed to be very low.

**Figure 9:**
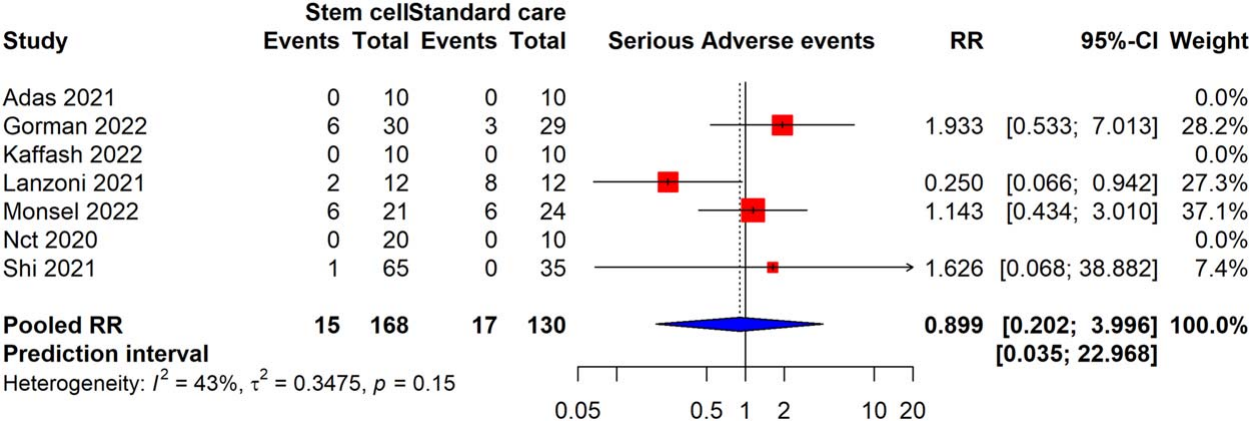
Forest plot detailing the rate of serious adverse events in COVID-19 patients undergoing stem cell therapy in contrast to those receiving standard care.

Regarding serious adverse events, data from seven studies with a total of 298 participants (168 receiving the intervention and 130 in the control group) revealed an RR of 0.899 (95% CI: 0.202–3.99). This result suggests that the intervention does not significantly increase or decrease the risk of serious adverse events in comparison to standard care, as indicated by the wide CI that spans both below and above the null value of 1. The heterogeneity for this outcome was moderately high (*I* ^2^=43%), with evident variation in how the intervention affects serious adverse events across various studies, indicating differences in outcomes related to the intervention’s safety and efficacy (Fig. 9). The certainty of evidence was found to be very low. The publication bias assessment is given in Supplementary Figure S1 (Supplemental Digital Content 1, http://links.lww.com/JS9/D45).

## Discussion

This umbrella review has meticulously consolidated the existing data on the safety and effectiveness of stem cell therapy for COVID-19, offering a detailed examination of its potential benefits and limitations, particularly MSCs, for treating COVID-19. The current global health crisis has necessitated the exploration of innovative therapeutic methods to reduce the severe COVID-19 manifestations, such as ARDS. The results of our comprehensive meta-analyses, derived from 24 systematic reviews, suggest that stem cell therapy, while promising, presents a complex therapeutic landscape with varying degrees of efficacy across different clinical outcomes. The most significant finding of our review is the potential reduction in mortality rates associated with stem cell therapy in COVID-19 patients. This result, supported by a low level of evidence, is encouraging and underscores the need for further large-scale, high-quality RCTs to substantiate these preliminary findings. The findings suggesting reduced hospital stay lengths and decreased reliance on invasive ventilation highlight promising outcomes of stem cell therapy in COVID-19 treatment, with moderate certainty, suggesting that stem cell therapy may facilitate a quicker recovery and reduce the burden on critical healthcare resources. However, the findings regarding the reduction in CRP levels, improvement in symptom remission rates, and the lack of significant effect on the SpO_2_/FiO_2_ ratio illustrate the intricacy of the immune reaction in COVID-19 and the multifaceted nature of stem cell therapy. The very low to low certainty of evidence for these outcomes reflects the need for cautious interpretation and suggests that while stem cell therapy has potential anti-inflammatory effects, its role in directly improving lung function and oxygenation in COVID-19 patients remains uncertain. Despite this, the consistency in the direction of the effects across studies suggests a potential therapeutic role for stem cells in COVID-19 treatment. The moderate certainty of evidence for outcomes such as reduced hospital stay and decreased need for invasive ventilation is particularly noteworthy, as these indicators have significant implications for patient outcomes and healthcare system strain. It is important to highlight the relatively safe profile of stem cell therapy as indicated by the analysis of adverse events. There was no observed significant difference in the occurrence of adverse and serious adverse events between patients receiving stem cell therapy and those given standard care. These findings suggest that MSC therapy could be a safe option for COVID-19 patients. This is an important consideration, as the introduction of any new therapy must balance efficacy with safety.

In our analysis, certain outcomes, notably CRP levels, demonstrated considerable heterogeneity (*I*²=89%). This high level of heterogeneity prompts further investigation into its potential sources. It is crucial to consider various factors that could contribute to this variation, including differences in study design, patient demographics, baseline severity of COVID-19, and specific stem cell protocols used across studies. Furthermore, the timing of intervention and the dosage of stem cells administered could also influence the inflammatory response differently in different cohorts. The level of evidence for some outcomes was categorized as low or very low. This categorization is due to the imprecision of results, which can be attributed to the small sample sizes and the overlapping of CI with the point of no effect.

The clinical implications of this umbrella review are profound, suggesting that MSC therapy holds potential as a therapeutic avenue for patients with COVID-19, particularly in severe cases where conventional treatments may fall short. The observed reduction in mortality rates and the need for invasive ventilation, coupled with the safety profile evidenced by the lack of increase in adverse events, position MSC therapy as a promising candidate for integrative treatment strategies^23^. Clinical practitioners need to consider the potential of MSC therapy within the context of individual patient profiles and the broader clinical management of COVID-19, especially as the disease evolves and new variants emerge^66^. Our findings suggest that MSC therapy shows considerable promise particularly in severe cases of COVID-19 characterized by ARDS and cytokine storm. The ability of MSCs to modulate immune response and promote tissue regeneration makes them particularly suitable for patients experiencing severe inflammatory responses. Moreover, patients who are at a higher risk of progressing to mechanical ventilation or those already requiring respiratory support may benefit significantly from MSC treatments. This therapy could potentially reduce the inflammation and improve lung function, which is critical in severe cases.

Looking to the future, research should aim to elucidate the mechanistic underpinnings of how MSCs exert their effects in the context of viral-induced inflammatory responses. Large-scale, multicentric RCTs with longer follow-up periods are needed to validate the effectiveness and safety of MSC therapy over extended timeframes. Additionally, future studies should strive to determine the optimal dosing, timing, and methods of stem cell administration to maximize therapeutic outcomes. Given the heterogeneity observed in some outcomes, further investigation is also required to identify, which subsets of patients might benefit most from MSC therapy. As the pandemic progresses with new variants for the virus, evaluating the use of MSC therapy in the context of emerging COVID-19 variants and in patients with long COVID symptoms will be crucial for developing comprehensive care protocols that address the full spectrum of the disease’s impact^67–69^. Our results endorse further research into stem cell therapy for COVID-19 treatment, with an emphasis on conducting high-quality research to fully understand its potential benefits and limitations. While the world still contends with the ongoing pandemic and its aftermath, the exploration of innovative treatments such as stem cell therapy is imperative^65,70–73^. Future research should aim to refine the selection of patient populations, standardize outcome measures, and improve the methodological rigor of studies to build upon the tentative foundation laid by the current body of evidence.

Our study has several strengths, including the synthesis of evidence from all available studies that encompass a wide array of clinically important outcomes. Our review team was comprised of multidisciplinary experts, enhancing the robustness of our analysis. We adhered to PRISMA guidelines (Supplemental Digital Content 1, http://links.lww.com/JS9/D45) and followed the JBI methodology for this umbrella review, ensuring a structured and systematic approach to our research. Additionally, we were able to determine the level of available evidence with the GRADE criteria. However, there are limitations to our study. The certainty of evidence for some outcomes was low, mostly due to the small overall sample size. We were unable to assess publication bias for several outcomes owing to the limited number of available studies. Furthermore, there was variability in the types of patients and their existing comorbidities across trials, which may affect the generalizability of the findings. Future studies are required for a better understanding of the benefits of stem cell therapy in treating diseases like COVID-19, with a focus on larger and more diverse patient populations to strengthen the evidence base.

## Conclusion

In our analysis, we synthesized evidence from a substantial collection of studies that encompass a broad spectrum of outcomes related to stem cell therapy. Overall, it has been observed that stem cell therapy is beneficial in treating COVID-19. The data suggest a promising direction for the use of MSC therapy in reducing mortality and morbidity associated with the disease. Future research should focus on large-scale randomized trials to consolidate the evidence base, optimizing dosing, timing, and methods to fully explore the potential of stem cell therapy in the clinical management of COVID-19.

## Ethical approval

Not applicable.

## Consent

Not applicable.

## Source of funding

The authors received no funding.

## Author contribution

C.T., M.N.K., and S.A.: conceptualization; M.A.A. and M.S.A.-T.: data curation; A.D.: formal analysis; N.J.A.F., N.A.A.-Z., and M.J.A.M.: investigation; S.R., P.S., and L.T.: methodology; A.A.R., T.Y.A.S., and P.R.P.: project administration; K.A.A. and S.H.A.-A.: resources; A.A.R.: software; A.H. and J.A.: supervision; Q.S.Z.: validation; M.S.A.-T.: visualization.

## Conflicts of interest disclosure

The authors declare no conflict of interest.

## Research registration unique identifying number (UIN)

PROSPERO: CRD42024507255.

## Guarantor

Prakasini Satapathy.

## Data availability statement

The data are available in the supplementary material and with the authors, available upon request.

## Provenance and peer review

Invited.

## Supplementary Material

SUPPLEMENTARY MATERIAL
