# Supplementary material for: Stem cell therapy for COVID-19 treatment: an umbrella review
Source: Int J Surg. 2024 Jul 5;110(10):6402–17. doi: 10.1097/JS9.0000000000001786 (PMC11487013; doi:10.1097/JS9.0000000000001786)
Supplement: SUPPLEMENTARY MATERIAL [file js9-110-6402-s001.docx]

**SUPPLEMENTARY MATERIALS**

**
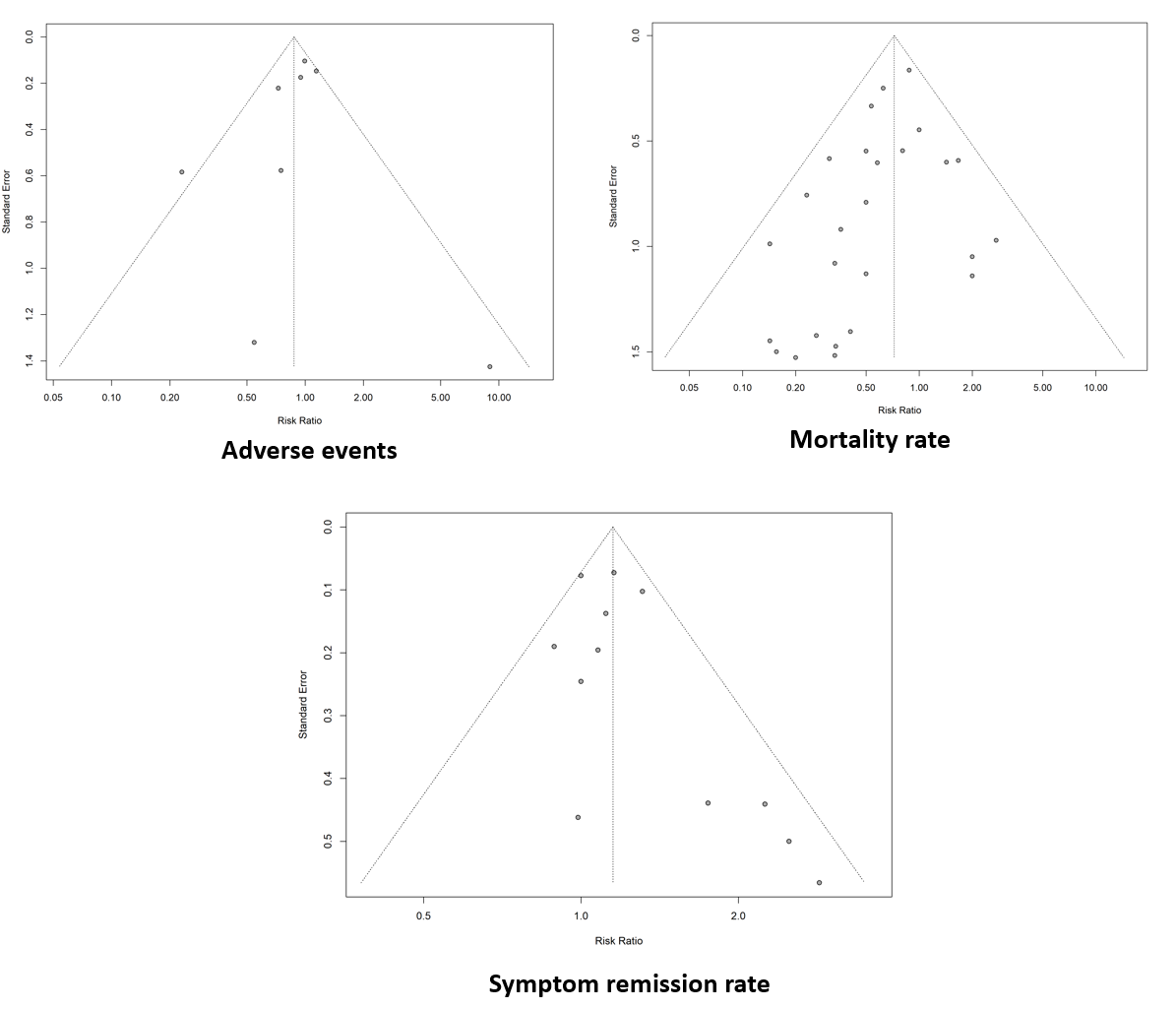
**

**Figure S1.**  Funnel plots depicting the publication bias.

## **Table S1.** PRISMA Checklist

| **Section and Topic** | **Item #** | **Checklist item** | **Location where item is reported** |
| --- | --- | --- | --- |
| **TITLE** | | |  |
| Title | 1 | Identify the report as a systematic review. | 1 |
| **ABSTRACT** | | |  |
| Abstract | 2 | See the PRISMA 2020 for Abstracts checklist. (made as per the Journal guidelines) | 2 |
| **INTRODUCTION** | | |  |
| Rationale | 3 | Describe the rationale for the review in the context of existing knowledge. | 3 |
| Objectives | 4 | Provide an explicit statement of the objective(s) or question(s) the review addresses. | 3 |
| **METHODS** | | |  |
| Eligibility criteria | 5 | Specify the inclusion and exclusion criteria for the review and how studies were grouped for the syntheses. | 4 |
| Information sources | 6 | Specify all databases, registers, websites, organisations, reference lists and other sources searched or consulted to identify studies. Specify the date when each source was last searched or consulted. | 4 |
| Search strategy | 7 | Present the full search strategies for all databases, registers and websites, including any filters and limits used. | Table S3 |
| Selection process | 8 | Specify the methods used to decide whether a study met the inclusion criteria of the review, including how many reviewers screened each record and each report retrieved, whether they worked independently, and if applicable, details of automation tools used in the process. | 4 |
| Data collection process | 9 | Specify the methods used to collect data from reports, including how many reviewers collected data from each report, whether they worked independently, any processes for obtaining or confirming data from study investigators, and if applicable, details of automation tools used in the process. | 4, 5 |
| Data items | 10a | List and define all outcomes for which data were sought. Specify whether all results that were compatible with each outcome domain in each study were sought (e.g., for all measures, time points, analyses), and if not, the methods used to decide which results to collect. | 4, Table 1 |
|  | 10b | List and define all other variables for which data were sought (e.g., participant and intervention characteristics, funding sources). Describe any assumptions made about any missing or unclear information. | 4 |
| Study risk of bias assessment | 11 | Specify the methods used to assess risk of bias in the included studies, including details of the tool(s) used, how many reviewers assessed each study and whether they worked independently, and if applicable, details of automation tools used in the process. | 5, Table S4 |
| Effect measures | 12 | Specify for each outcome the effect measure(s) (e.g. risk ratio, mean difference) used in the synthesis or presentation of results. | 5 |
| Synthesis methods | 13a | Describe the processes used to decide which studies were eligible for each synthesis (e.g. tabulating the study intervention characteristics and comparing against the planned groups for each synthesis (item #5)). | 5, Table 1 |
|  | 13b | Describe any methods required to prepare the data for presentation or synthesis, such as handling of missing summary statistics, or data conversions. | NA |
|  | 13c | Describe any methods used to tabulate or visually display results of individual studies and syntheses. | 4 |
|  | 13d | Describe any methods used to synthesize results and provide a rationale for the choice(s). If meta-analysis was performed, describe the model(s), method(s) to identify the presence and extent of statistical heterogeneity, and software package(s) used. | 5 |
|  | 13e | Describe any methods used to explore possible causes of heterogeneity among study results (e.g. subgroup analysis, meta-regression). | 6 |
|  | 13f | Describe any sensitivity analyses conducted to assess robustness of the synthesized results. | 6 |
| Reporting bias assessment | 14 | Describe any methods used to assess risk of bias due to missing results in a synthesis (arising from reporting biases). | NA |
| Certainty assessment | 15 | Describe any methods used to assess certainty (or confidence) in the body of evidence for an outcome. | NA |
| **RESULTS** | | |  |
| Study selection | 16a | Describe the results of the search and selection process, from the number of records identified in the search to the number of studies included in the review, ideally using a flow diagram. | Figure-1 |
|  | 16b | Cite studies that might appear to meet the inclusion criteria, but which were excluded, and explain why they were excluded. | 6, Table 1 |
| Study characteristics | 17 | Cite each included study and present its characteristics. | Table-1 |
| Risk of bias in studies | 18 | Present assessments of risk of bias for each included study. | Table S4 |
| Results of individual studies | 19 | For all outcomes, present, for each study: (a) summary statistics for each group (where appropriate) and (b) an effect estimate and its precision (e.g. confidence/credible interval), ideally using structured tables or plots. | Table 1, Figure 2,3 |
| Results of syntheses | 20a | For each synthesis, briefly summarise the characteristics and risk of bias among contributing studies. | 5 |
|  | 20b | Present results of all statistical syntheses conducted. If meta-analysis was done, present for each the summary estimate and its precision (e.g. confidence/credible interval) and measures of statistical heterogeneity. If comparing groups, describe the direction of the effect. | 5, 6 Figure 2, 3 |
|  | 20c | Present results of all investigations of possible causes of heterogeneity among study results. | 6 |
|  | 20d | Present results of all sensitivity analyses conducted to assess the robustness of the synthesized results. | NA |
| Reporting biases | 21 | Present assessments of risk of bias due to missing results (arising from reporting biases) for each synthesis assessed. | NA |
| Certainty of evidence | 22 | Present assessments of certainty (or confidence) in the body of evidence for each outcome assessed. | NA |
| **DISCUSSION** | | |  |
| Discussion | 23a | Provide a general interpretation of the results in the context of other evidence. | 6 |
|  | 23b | Discuss any limitations of the evidence included in the review. | 7 |
|  | 23c | Discuss any limitations of the review processes used. | 8 |
|  | 23d | Discuss implications of the results for practice, policy, and future research. | 9 |
| **OTHER INFORMATION** | | |  |
| Registration and protocol | 24a | Provide registration information for the review, including register name and registration number, or state that the review was not registered. | 4 |
|  | 24b | Indicate where the review protocol can be accessed, or state that a protocol was not prepared. | 4 |
|  | 24c | Describe and explain any amendments to information provided at registration or in the protocol. | NA |
| Support | 25 | Describe sources of financial or non-financial support for the review, and the role of the funders or sponsors in the review. | 9 |
| Competing interests | 26 | Declare any competing interests of review authors. | 9 |
| Availability of data, code and other materials | 27 | Report which of the following are publicly available and where they can be found: template data collection forms; data extracted from included studies; data used for all analyses; analytic code; any other materials used in the review. | 9 |

**Table S2.** Inclusion and Exclusion criteria

**Research Question: "** **Stem cell therapy for COVID-19 treatment”**

| **Inclusion** | | **Exclusion** |
| --- | --- | --- |
| **Participants** | Patients diagnosed with COVID-19 irrespective of age, gender, disease severity, or the presence of comorbidities. | Not diagnosed with COVID-19 |
| **Intervention** | Stem cell therapy in treating COVID-19 | No stem cell therapy |
| **Outcome** | Mortality rates, the necessity for mechanical ventilation, duration of hospital stays, improvements in lung function, and any reported adverse effects | NA |
| **Study Designs** | Systematic reviews and meta-analysis | Clinical trials,  Letter to editor,  Commentaries,  Abstract only, Case series, case reports, reviews, Discussion papers, animal studies |
|  | Date of Search- 15^th^ of February 2024  Published articles in English | Unavailable full-text articles |

**Table S3. The adjusted search terms as per searched electronic databases [as of 15 February 2024]**

| **Database** | **No** | **Search Query** | **Results** |
| --- | --- | --- | --- |
| **PubMed** | | | |
|  | #1 | ("systematic review"[All Fields] OR "meta-analysis"[All Fields] OR "metaanalysis"[All Fields]) AND (("acute respiratory distress syndrome"[All Fields] AND "COVID-19"[MeSH Terms]) OR "COVID-19"[All Fields] OR "coronavirus"[All Fields] OR "SARS-CoV-2"[All Fields]) AND ("stem cells"[MeSH Terms] OR "stem cell*"[All Fields] OR "stromal cell*"[All Fields] OR "mesenchymal cell*"[All Fields] OR "Wharton's Jelly cells"[All Fields] OR "mother cell"[All Fields] OR "progenitor cells"[All Fields] OR "bone marrow"[All Fields] OR "umbilical cord"[All Fields] OR "colony forming units"[All Fields]) | 135 |
| **Embase** | | | |
|  | #1 | ('stem cells'/exp OR 'stem cells' OR 'stromal cells'/exp OR 'stromal cells' OR 'wharton jelly cells' OR 'mother cell'/exp OR 'mother cell' OR 'mesenchymal stem cell'/exp OR 'mesenchymal stem cell' OR 'progenitor cells' OR 'bone marrow'/exp OR 'bone marrow' OR 'umbilical cord'/exp OR 'umbilical cord' OR 'colony forming units') AND ('covid-19'/exp OR 'covid-19' OR 'coronavirus'/exp OR 'coronavirus' OR 'sars-cov-2'/exp OR 'sars-cov-2') AND ('systematic review'/exp OR 'systematic review' OR 'meta-analysis'/exp OR 'meta-analysis') | 357 |
| **Web of Science** | | | |
|  | #1 | ALL=(("systematic review" OR "meta-analysis" OR "metaanalysis") AND (("acute respiratory distress syndrome" AND "COVID-19") OR "COVID-19" OR "coronavirus" OR "SARS-CoV-2") AND ("stem cells" OR "stem cell*" OR "stromal cell*" OR "mesenchymal cell*" OR "Wharton's Jelly cells" OR "mother cell" OR "progenitor cells" OR "bone marrow" OR "umbilical cord" OR "colony forming units")) | 130 |
| **Cochrane** | | | |
|  | #1 | (“stem cells” OR “stromal cells” OR “Wharton Jelly cells” OR “mother cell” OR "mesenchymal stem cell" OR “progenitor cells” OR “bone marrow” OR “umbilical cord” OR “colony forming units”) AND ("COVID-19" OR "COVID-19" OR "coronavirus" OR "SARS-CoV-2") AND ("Systematic review" or "meta-analysis") | 32 |

**Supplementary Table S4.** Summary of quality assessment of included systematic reviews using AMSTAR 2.

| **Study ID** | **1** | **2** | **3** | **4** | **5** | **6** | **7** | **8** | **9** | **10** | **11** | **12** | **13** | **14** | **15** | **16** |  |
| --- | --- | --- | --- | --- | --- | --- | --- | --- | --- | --- | --- | --- | --- | --- | --- | --- | --- |
|  | **n-critical domain** | **Critical domain** | **n-critical domain** | **Critical domain** | **n-critical domain** | **n-critical domain** | **Critical domain** | **n-critical domain** | **Critical domain** | **n-critical domain** | **Critical domain** | **n-critical domain** | **Critical domain** | **n-critical domain** | **Critical domain** | **n-critical domain** | **Overall** |
| Arabpour 2021 | Yes | Yes | Yes | Yes | Yes | Yes | No | Yes | Yes | No | Yes | Yes | No | No | Yes | Yes | Moderate |
| Cao 2022 | Yes | Yes | Yes | Yes | No | No | No | Yes | no | No | Yes | Yes | No | No | No | Yes | Low |
| Chen 2022 | Yes | Yes | Yes | Yes | Yes | Yes | No | Yes | Yes | No | Yes | Yes | Yes | no | No | Yes | Low |
| Chen 2023 | Yes | Yes | Yes | Yes | No | No | No | Yes | Yes | No | Yes | Yes | no | no | No | Yes | Critically low |
| Couto 2023 | Yes | Yes | Yes | Yes | Yes | Yes | No | Yes | No | No | Yes | Yes | No | No | Yes | Yes | Low |
| Cuevas-Gonzalez 2021 | Yes | Yes | Yes | Yes | Yes | Yes | No | no | Yes | No | No | No | No | No | No | Yes | Critically low |
| Javed 2022 | Yes | Yes | Yes | Yes | Yes | Yes | No | Yes | No | No | No | No | Yes | no | No | Yes | Critically low |
| Kandula 2023 | Yes | Yes | Yes | Yes | No | No | No | Yes | Yes | No | No | No | yes | No | No | Yes |  |
| Kirkham 2022 | Yes | No | Yes | Yes | Yes | Yes | No | Yes | Yes | No | Yes | Yes | Yes | No | No | Yes | Low |
| Kirkham 2022 (2nd) | Yes | Yes | Yes | Yes | Yes | Yes | No | Yes | Yes | No | Yes | Yes | No | No | Yes | Yes | Moderate |
| Kirkham 2023 | Yes | No | Yes | Yes | Yes | Yes | No | Yes | no | No | Yes | Yes | No | No | No | Yes | Low |
| Li 2023 | Yes | No | Yes | Yes | No | No | No | Yes | No | No | Yes | Yes | No | No | No | Yes | Low |
| Liu 2023 | Yes | No | Yes | Yes | Yes | Yes | No | Yes | Yes | No | Yes | Yes | No | No | No | Yes | Moderate |
| Qu 2022 | Yes | Yes | Yes | Yes | Yes | Yes | No | Yes | Yes | No | Yes | Yes | No | No | No | Yes | Low |
| Tamis 2023 | Yes | Yes | Yes | Yes | No | No | No | Yes | Yes | No | No | No | No | No | No | Yes | Critically low |
| Taufiq 2023 | Yes | no | Yes | Yes | Yes | Yes | No | Yes | Yes | No | Yes | yes | No | No | Yes | Yes | Low |
| Wang 2021 | Yes | No | Yes | Yes | Yes | Yes | No | Yes | Yes | No | Yes | No | No | No | No | Yes | Critically low |
| Wang 2023 | Yes | Yes | Yes | Yes | Yes | Yes | No | Yes | Yes | No | Yes | No | No | No | Yes | Yes | Low |
| Yan 2023 | Yes | Yes | Yes | Yes | Yes | Yes | No | Yes | Yes | No | Yes | No | Yes | no | Yes | Yes | Moderate |
| Yang 2023 | Yes | Yes | Yes | Yes | Yes | Yes | No | Yes | Yes | No | Yes | No | no | no | Yes | Yes | Low |
| Yao 2022 | Yes | No | Yes | Yes |  |  | No | Yes | Yes | No | Yes | No | No | No | Yes | Yes | Low |
| Zanirati 2021 | Yes | Yes | Yes | Yes | No | No | No | Yes | No | No | Yes | No | No | No | No | Yes | Critically low |
| Zhang 2022 | Yes | Yes | Yes | Yes | No | No | No | Yes | Yes | No | Yes | No | Yes | no | Yes | Yes | Low |
| Zhang 2023 | Yes | No | Yes | Yes | No | No | No | Yes | Yes | No | Yes | No | yes | No | Yes | Yes | Low |

| **Table S5. Stem cells therapy compared to standard care in COVID-19 patient**  **Bibliography:** | | | | | | | | | | | |
| --- | --- | --- | --- | --- | --- | --- | --- | --- | --- | --- | --- |
| **Certainty assessment** | | | | | | | **Summary of findings** | | | | |
| **Participants (studies) Follow-up** | **Risk of bias** | **Inconsistency** | **Indirectness** | **Imprecision** | **Publication bias** | **Overall certainty of evidence** | **Study event rates (%)** | | **Relative effect (95% CI)** | **Anticipated absolute effects** | |
|  |  |  |  |  |  |  | **With standard care** | **With Stem cells therapy** |  | **Risk with standard care** | **Risk difference with Stem cells therapy** |
| **Mortality** | | | | | | | | | | | |
| 1422 (33 RCTs) | serious^a^ | not serious | not serious | serious^b^ | none | ⨁⨁◯◯ Low | 193/765 (25.2%) | 112/657 (17.0%) | **RR 0.72** (0.59 to 0.88) | 252 per 1,000 | **71 fewer per 1,000** (from 103 fewer to 30 fewer) |
| **CRP level (assessed with: SMD)** | | | | | | | | | | | |
| 220 (6 RCTs) | not serious | serious^c^ | not serious | serious^b^ | none | ⨁⨁◯◯ Low | 113 | 107 | - | - | SMD **1.198 SD lower** (2.591 lower to 0.195 higher) |
| **Symptom remission rate (assessed with: RR)** | | | | | | | | | | | |
| 724 (12 RCTs) | serious^a^ | not serious | not serious | very serious^b,d^ | none | ⨁◯◯◯ Very low | 164/353 (46.5%) | 201/371 (54.2%) | **RR 1.151** (0.996 to 1.330) | 465 per 1,000 | **70 more per 1,000** (from 2 fewer to 153 more) |
| **length of hospital stay (assessed with: Mean number of days)** | | | | | | | | | | | |
| 269 (5 RCTs) | not serious | not serious | not serious | serious^b^ | none | ⨁⨁⨁◯ Moderate | 134 | 135 | - | The mean length of hospital stay was **0** Days | MD **4 Days lower** (4.68 lower to 3.33 lower) |
| **Adverse events (assessed with: RR)** | | | | | | | | | | | |
| 860 (17 RCTs) | serious^a^ | not serious | not serious | serious^b,d^ | none | ⨁⨁◯◯ Low | 118/414 (28.5%) | 125/446 (28.0%) | **RR 0.870** (0.607 to 1.265) | 285 per 1,000 | **37 fewer per 1,000** (from 112 fewer to 76 more) |
| **Serious adverse events** | | | | | | | | | | | |
| 298 (7 RCTs) | not serious | not serious | not serious | very serious^b,e^ | none | ⨁⨁◯◯ Low | 17/130 (13.1%) | 15/168 (8.9%) | **RR 0.899** (0.202 to 3.996) | 131 per 1,000 | **13 fewer per 1,000** (from 104 fewer to 392 more) |
| **Need for mechanical invasive ventilation (assessed with: RR)** | | | | | | | | | | | |
| 328 (4 RCTs) | not serious | not serious | not serious | serious^b^ | none | ⨁⨁⨁◯ Moderate | 61/223 (27.4%) | 13/105 (12.4%) | **RR 0.521** (0.320 to 0.847) | 274 per 1,000 | **131 fewer per 1,000** (from 186 fewer to 42 fewer) |
| **Time for symptom improvement (assessed with: MD)** | | | | | | | | | | | |
| 99 (2 RCTs) | not serious | not serious | not serious | serious^b^ | none | ⨁⨁◯◯ Low | 58 | 41 | - | The mean time for symptom improvement was **0** | MD **4.01 lower** (6.33 lower to 1.68 lower) |
| **SpO2/FiO2 (assessed with: WMD)** | | | | | | | | | | | |
| 163 (4 RCTs) | not serious | serious^c^ | not serious | very serious^b,d^ | none | ⨁◯◯◯ Very low | 83 | 80 | - | The mean spO2/FiO2 was **0** SD | WMD **4.29 SD lower** (22.26 lower to 30.6 higher) |
