# Supplementary figures and images for: Stem cell therapy for COVID-19 treatment: an umbrella review
Source: Int J Surg. 2024 Jul 5;110(10):6402–17. doi: 10.1097/JS9.0000000000001786 (PMC11487013; doi:10.1097/JS9.0000000000001786)

**
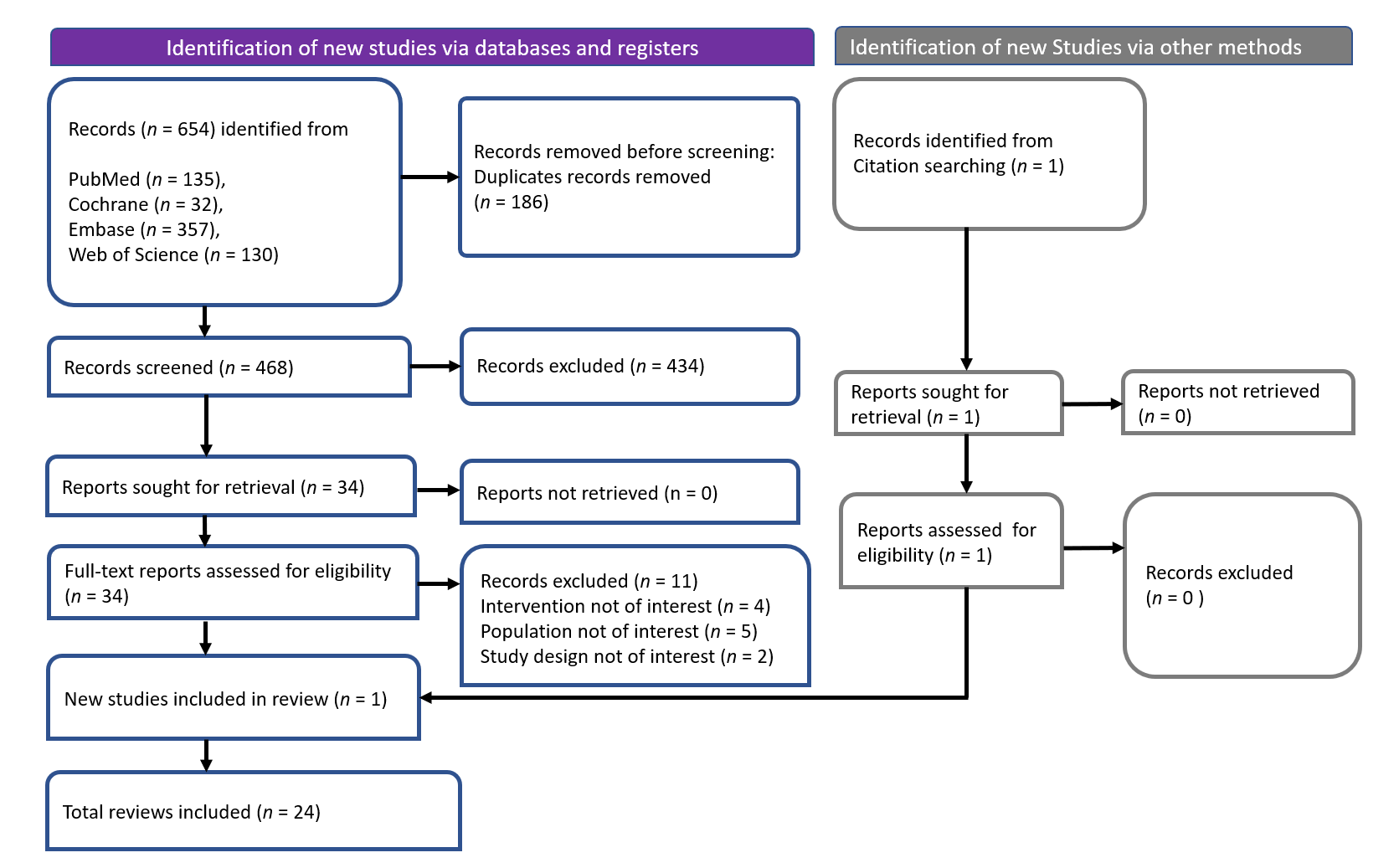
**

**Figure 1.** PRISMA flowchart illustrating the process of article screening and selection.

Supplement: SUPPLEMENTARY MATERIAL [file js9-110-6402-s002.docx]
